# A Current Management Strategy for Children with Chronic Viral Hepatitis B, Based on International and National Guidelines

**DOI:** 10.34763/jmotherandchild.20232701.d-23-00006

**Published:** 2023-08-31

**Authors:** Inna M. Nesina, Tetyana O. Kryuchko, Olha A. Poda, Olha Ya. Tkachenko, Nataliia V. Kuzmenko, Liudmyla M. Bubyr

**Affiliations:** Department of Pediatrics No. 2, Poltava State Medical University, Poltava, Ukraine

**Keywords:** children, hepatitis B virus, chronic hepatitis B, diagnosis, treatment, management, prevention

## Abstract

**Background:**

Peculiarities of the course of chronic viral hepatitis B in children cause an important medical and social problem of health care, despite the implementation of modern treatment and prevention protocols. Pathogenetic mechanisms of the development and progression of viral hepatitis B infection, the presence of occult poorly diagnosed form, the impossibility of completely eliminating the virus and the specificity of the immune response in children are still not fully solved scientific problems.

**Material and Methods:**

The aim of this review is to examine current strategies for the diagnosis and treatment of chronic hepatitis B in children, based on international and national guidelines.

**Results:**

A detailed analysis of modern guidelines on the course and pathogenesis of viral hepatitis B infection confirms the fact that chronic hepatitis B is characterised by a complex interaction between the immune system of the virus and the patient, whose dynamic balance is not only responsible for the various phases of chronic viral hepatitis B infection but also leads to the result of antiviral treatment.

**Conclusion:**

Despite the introduction of vaccination of children against hepatitis B, the level of viral hepatitis B vaccination of children in Ukraine remains insufficient, which leads to the further spread of the infection. Currently available antiviral drugs can provide functional treatment of viral hepatitis B infection in a limited number of patients, but today's Ukrainian realities have caused a change in approach to the treatment and monitoring of patients, which may negatively affect the implementation of the key goals of the World Health Organization Global Strategy on the prevention, diagnosis and treatment of viral hepatitis.

## Introduction

Chronic hepatitis B (CHB) has undoubtedly remained a global public health problem in recent decades, despite the introduction of modern treatment and prevention protocols. According to the World Health Organization (WHO) Global Report on Viral Hepatitis [1,2], the disease causes about 1.34 million deaths annually. WHO estimates that in 2021, 296 million people will have chronic HBV infection, including six million children under five years of age, with 1.5 million new infections each year. Despite widespread HBV infection, access to diagnosis and treatment remains poor – only 9% of people with chronic HBV and 20% of those with chronic viral hepatitis C (HCV) worldwide are aware of their diagnosis, with only 8% of those with chronic HBV and 7.4% of those with chronic HCV, respectively, able to access treatment.

To reduce the contribution of viral hepatitis to morbidity and mortality worldwide since 2016, the WHO has adopted the Global Strategy for combating these diseases during 2016–2021 [3,4,5]. The ultimate goal of the Strategy is to reduce mortality from chronic viral hepatitis B and C to 10% by 2020 and to 65% by 2030 (from the current 1.4 million deaths to 0.5 million for both diseases).

Surveys by Ukrainian epidemiologists have shown that the incidence of viral hepatitis in Ukraine is 7–9% of the country's total population, while in European countries this figure ranges from 0.2% to 2.5%. Overall, given the specific nature of the disease, the true number of viral hepatitis patients in Ukraine could be much higher. In November 2019, Ukraine joined the Global Strategy for the elimination of viral hepatitis B and C by adopting the State Strategy for combating HIV/AIDS, tuberculosis, and viral hepatitis until 2030. The Strategy identifies key goals and targets aimed at eliminating HBV as a public health threat. According to the Strategy's 2030 targets, 90% of people with HBV should be identified and treated [6].

## Objective

The aim of this review is to examine current strategies for the diagnosis and treatment of CHB in children, based on international and national guidelines.

CHB is a dynamic disease with an unpredictable course. For a long time, the assumption that those infected at birth or in early childhood enter a phase of long-term immune tolerance with little or no disease progression over two to three decades has been increasingly challenged. The concept of immune tolerance of the disease has not been scientifically validated so far, as there is a lack of clinically relevant immunological indicators for this cohort of patients [7].

In recent decades, it has been established that HBV is not directly cytopathic and that HBV results from the persistence of a defective immune response, making it impossible to control the virus. This is supported by the fact that one in three people has been exposed to HBV at least once in their life, but many of these individuals developed natural immunity to infection [8,9,10], yet an inadequate immune response is common in 10% of adults and almost all infants infected at birth, all of which result in lifelong chronic HBV infection. An unresolved problem is the ability of the hepatitis B virus, even after loss of HBsAg, to persist in some hepatocytes in the form of a viral mini-chromosome superspiralised covalently closed HBV DNA (cccDNA) reactivation of viral replication when immune competence is compromised [11,12,13,14].

Thus, the course of HBV infection is determined by a complex interaction between the immune system of the virus and the infected person, the dynamic equilibrium of which is responsible not only for the different phases of chronic HBV infection, but also influences the outcome of antiviral treatment. With this in mind, it can be said that the rate of response to therapy depends on the phase of infection (HBeAg positive or HBeAg negative/anti-HBe positive), viral load and disease activity [13,15].

It is important enough recognise clearly that the prevalence of HBV infection in children depends significantly on the vaccination of infants at birth. Up to 30% of HBV infection can be attributed to horizontal transmission in early childhood within the family among unvaccinated children [13,14,16]. Although vaccination in Ukraine began only after 2000, meaning that almost all adults in the country have no protection against infection, the level of HBV vaccination of children remains inadequate, leading to further spread of infection. According to the Ukrainian Centre for Public Health, about 2.5 million children do not have immune protection against hepatitis B, with 1.1 million of those children under five years of age, which is the most vulnerable cohort, as 95% of chronic HBV infections develop in this age group.

With the reform of the medical sector in Ukraine, the problem of CHB in children is gaining new momentum due to important aspects of the course of the disease, insufficient identification of those infected, lack of a unified registry of infected as a mechanism to control infection, likelihood of infection in newborns and lack of opportunity for medication eradication of the virus. Following Ukraine's accession to the Global Strategy, the development of new standards became an urgent need, as the unified clinical protocols for primary, secondary (specialised) and tertiary (highly specialised) medical care for patients with viral hepatitis B and C, adopted in 2016, had lost relevance, in particular due to the availability of complex diagnostic approaches and the emergence of the latest direct acting antivirals drugs.

In addition, the development of new standards of medical care was driven by the need to approve simplified diagnostic algorithms for viral hepatitis. It is planned that the changes in the Standards will ensure earlier detection of patients with viral hepatitis disease, reduce the number of patients lost from follow-up and facilitate the immediate initiation of treatment. A separate emphasis in the new Standards is placed on measures to prevent viral hepatitis infections and the definition of a list of children recommended for testing: a list of persons who should be tested for viral hepatitis B is defined, and it is stated that all individuals should be tested for HCV at least once in their life and repeated regularly, if there are risk factors. The new Standards abolish outdated unified clinical protocols recommending the use of outdated treatment regimens (pegylated interferons) and a complex screening algorithm. According to the goals of State Strategy on HIV/AIDS, tuberculosis, and viral hepatitis until 2030, approved by the Cabinet of Ministers of Ukraine № 1415-r of 27 November 2019, 50% of people with HBV should be diagnosed by 2025, with 90% of such people diagnosed by 2030 [5].

The Adapted Clinical Guideline for Hepatitis B is based on the “Clinical Practice Guidelines on the management of hepatitis B virus infection, EASL 2017” [17] and the “Guidelines for the prevention, care and treatment of persons with chronic hepatitis B infection” [35] (2015, WHO), with additional sections from the following sources: “Update on prevention, diagnosis, and treatment of chronic hepatitis B: AASLD 2018 hepatitis B guidance” [9], “Asian-Pacific clinical practice guidelines on the management of hepatitis B: 2015 update” [34], “Hepatitis B (chronic): diagnosis and management. Clinical guideline NICE, 2013” [36], “Management of chronic hepatitis B in childhood: ESPGHAN clinical practice guidelines: consensus of an expert panel on behalf of the European Society of Pediatric Gastroenterology, Hepatology and Nutrition” [37].

According to the recent recommendations of the European Association for the Study of Liver Diseases (EASL), a new nomenclature for HBV infection has been adopted, as it is recognised that the phase names previously used are not supported by immunological data and are not determinative in deciding the indication for antiviral therapy [17]. The nomenclature is based on the description of the two main characteristics of chronicity: infection/hepatitis (Table 1).

**Table 1.** Phases in natural history of chronic hepatitis B virus infection

**HBV phases**	HBeAg+infection^*^/Immune-tolerant phase^**^	HBeAg+ hepatitis^*^/Immune-active phase^**^	HBeAg−infection^*^/Inactive carrier/immune-control phase^**^	HBeAg−hepatitis^*^/Immune-escape phase^**^	HBsAg−infection^*^/Occult HBV infection (anti-HBc-positive)^**^

**Indicators**					
Serum HBV DNA	>10 · IU/ml	>2000 IU/ml (constantly raised or fluctuating)	<2000 IU/mL	>2000 IU/mL	undetectable
ALT	normal	elevated	normal	elevated	normal
HBeAg	positive	positive	negative	negative	negative
HBsAg	high	high/intermediate	low	intermediate	negative
cccDNA	+++++	+++++	++	+++	+
Integrated HBV DNA	+++	++++	+++	++++	+
Liver disease (Necro-inflammatory changes)	none/minimal	moderate to severe	none	moderate to severe	none
Progression to cirrhosis	none	possible	none	more rapid than in other phases	none
Treatment	not generally indicated	may be indicated	not indicated	may be indicated	not indicated

* New terminology

** Old terminology

Classification of the infection phase requires consistent monitoring of HBeAg, HBV DNA and serum alanine aminotransferase (ALT) levels. Importantly, the phases of chronic HBV infection may not necessarily be consecutive. The rate of spontaneous elimination of HBeAg is very low in this phase. Patients are significantly infectious in the first phase due to high levels of HBV DNA [14,17]. The outcome of this phase varies: most patients can achieve seroconversion of HBeAg to HBeAb and reduction of HBV DNA with progression to HBeAg-negative infection; in other patients, HBV replication may not be controlled, and the process will progress to HBeAg-negative phase of CHB for many years [14,17]. Patients in this phase 3 have a low risk of progression to cirrhosis or hepatocellular carcinoma, but there is progression to CHB, which is usually seen in HBeAg-negative patients. Disappearance of HBsAg and/or its seroconversion to HBsAb can occur spontaneously (1–3% per year). These patients usually have low (<1000 IU/mL) serum HBsAg levels [9,14,17]. This phase 4 is associated with a low rate of spontaneous remission of the disease. Most of these patients have HBV mutations in the pre-core and/or core promoter region of the nucleus, which excludes or minimises HBeAg expression [9,14]. This phase 5 elimination of HBsAg by the beginning of cirrhosis is associated with a minimal risk of cirrhosis, decompensation and hepatocellular carcinoma, and improved survival. However, if cirrhosis developed before HBsAg elimination, patients are still at risk for hepatocellular carcinoma, so follow-up should continue to prevent its formation in time. In these patients, HBV reactivation may lead to immunosuppression [9,14,17].

Given that the course and phases of HBV infection in children have not been well described and only a few studies, including seven large studies (90 patients, follow-up more than 10 years) [17], it can be stated that HBV infection in children has an acquired (perinatally or in early childhood) character, representing a phase with high replication and low inflammation, which is considered to last for several decades. It is worth noting that a 29-year prospective study in Italy showed that of 91 HBeAg-positive children, 89 had HBeAg to HBeAb seroconversion at around 30 years of age, with active CHB diagnosed in 1–5% of HBeAg-positive children in adolescence [18–19]. Long-term paediatric studies with children treated showed that the prevalence of cirrhosis and hepatocellular carcinoma was 0.2% and 0.5% in Taiwan [20], 2.7% and 0% in the UK, 0.6% and 0.6% in Greece, 3.6% and 1.8% in Italy, 3.8% and 0% in Romania, 0.8% and 0.4% in Canada and 0% and 1.5% in Japan, respectively [21].

The aims of antiviral therapy in both adults and children with chronic HBV infection are effective and sustained suppression of HBV replication to reduce the risk of cirrhosis and hepatocellular carcinoma [14,15,17, 22,23].

Ukrainian realities show that primary care physicians are charged with the main task of the WHO Global Strategy for Hepatitis Disease Control – screening and primary diagnosis of HBV and HCV infections. According to the listed recommendations, the diagnosis of HBV infection in children over 12 months of age and adolescents begins with the detection of HBsAg in serum by a serological test that meets the quality standard. The HBsAg test can be repeated at least six months after the first positive test to confirm chronic HBV infection. It is difficult to test infected children in the first six months of life because HBV DNA and HBsAg may be detected temporarily at birth or in the first few months of life and may not reflect chronic HBV infection. Children should be tested for HBsAg at 6 to 12 months of age to reduce the risk of false-positive results [24]. Thus, according to international protocols, the initial evaluation of patients with chronic HBV infection includes: a detailed history; physical examination; assessment of the activity and severity of liver damage: biochemical parameters (ALT, AST; gamma-glutamyl transpeptidase; alkaline phosphatase; bilirubin; serum albumin; gamma-globulin, total blood count; prothrombin time); evaluation of instrumental methods (ultrasound of the liver and other abdominal organs; histological examination of liver biopsy or noninvasive tests to determine disease activity (elastography) testing for all markers of HBV infection; HBV genotype identification is not necessary but may be useful in selecting patients for interferon-alpha therapy; screening to rule out co-morbidities, including autoimmune, metabolic liver disease, steatosis or steatohepatitis and other causes of chronic liver disease, including co-infections with hepatitis D virus, hepatitis C virus, and HIV; and hepatitis A virus (HAV). All of these host factors can be determined simply by a blood test except the fibrosis stage and steatosis grade, which are classically assessed through liver biopsy. Because of the limitations of biopsy (sampling error, cost and adverse events), non-invasive biomarkers have been developed. The most validated biomarkers, such as FibroTest and Fibroscan, are widely used and have already been validated as alternatives to biopsy in several countries [25]. There is controversy about the use of elastography in adolescents and children with chronic HBV infection – it is considered to be unvalidated, but studies evaluating elastography in children with other chronic liver diseases have shown its reliability in determining the stages of fibrosis [26].

According to current strategies, the final points of therapy are the induction of long-term HBV DNA reduction and elimination of HBeAg with or without seroconversion to HBeAb in HBeAg-positive patients; normalisation of ALT levels; and elimination of HBsAg with or without seroconversion to HBsAb (‘functional treatment’, indicating profound inhibition of HBV replication and viral protein expression).

The indications for treatment of adult patients with HBV infection were formulated by the European Association for the Study of the Liver (EASL) in 2017. For children, in contrast to adults, there are few recommendations on the optimal timing and indications for treatment. There are recommendations from the European Society for Paediatric Gastroenterology Hepatology and Nutrition and the American Association for Study of Liver Diseases (AASLD) [9,14]. Consequently, all the above guidelines recommend that the following should be assessed before starting treatment: stage of liver disease; HBV DNA concentration and ALT level; HBeAg status; family history of hepatocellular carcinoma, co-infection with HIV and other liver diseases.

Current guidelines recommend treatment of the following patients regardless to age: presence of cirrhosis; active hepatitis (HBeAg positive or HbeAg negative) with elevated levels of ALT and HBV DNA and histological signs of inflammation and fibrosis. AASLD specifically recommends treatment of HBeAg-positive adolescents and children with elevated ALT levels and identified HBV DNA without specifying the duration of the enzyme increase (most studies are based on an ALT increase of more than 1.3-times above the upper limit of normal for ≥6 months) [14]. According to the ESPGHAN guidelines, treatment is initiated when there is a sustained increase in ALT levels for at least six months in HBeAg-positive adolescents and children or for at least 12 months in HBeAg-negative adolescents and children with at least moderate inflammation and fibrosis as detected by histological examination of liver tissue [27]. And the Asian Pacific Association for the Study of the Liver (APASL) treatment option is closer to the EASL guidelines and recommends treatment of HBeAg-positive adolescents and children with HBV DNA concentrations >20 000 IU/mL and ALT levels more than twice the upper limit of normal for more than 12 months, and HBeAg-negative hepatitis with ALT levels more than twice the upper limit of normal for HBV DNA >2000 IU/mL [14]. A family history of hepatocellular carcinoma is an additional factor determining the indication for treatment (ESPGHAN, APASL). All major scientific international societies (ESPGHAN, AASLD, APASL, EASL) clearly agree on the treatment of both adults and children with fulminant or severe acute HBV infection; HBsAg-positive patients receiving immunosuppressive therapy or chemotherapy; HBsAg-positive patients who are indicated for liver transplantation and patients receiving transplants from HBcAb-positive donors [8, 14, 28, 29, 30, 34]. However, ESPGHAN recommends that the duration of antiviral treatment should be at least six months after discontinuation of immunosuppressive, cytotoxic and biological therapy, and if the risk of viral reactivation is high, treatment should be continued for at least 12 months.

The following drugs are currently used to treat adult patients with CHB: interferons; pegylated interferon (PegIFN-α); antiviral drugs: with low HBV resistance barrier: (lamivudine, adefovir dipivoxil, telbivudine; with high HBV resistance barrier: entecavir, tenofovir disoproxil fumarate, tenofovir alafenamide. Nucleos(t)ide analogues, which are available for treatment HBV infections, allow one to achieve elimination of HBsAg and seroconversion to HBsAb in less than 1% of adults and 1–6% of children. Increasing the duration of therapy increases the rate of HBsAg seroconversion [31,32,33].

EASL, AASLD and WHO recommend tenofovir or entecavir as the best initial therapy. Interferon-alpha preparations are considered second-line therapy by APASL, EASL and AASLD [9,27,33]. Interferon-alpha is approved by the US Food and Drug Administration and the European Medicines Agency (EMA) for the treatment of chronic HBV infection in children from one year old and older. PegIFN-α-2b is approved by the EMA in 2017; and for use in children over three years of age. Entecavir is recommended by WHO, EMA for ages two years and older, in Ukraine also from two years of age (if weighing at least 32.6 kg) and older. Tenofovir disoproxil fumarate was approved by EMA in 2019 for children ages two years and older, in Ukraine from 12 years and older. Tenofovir alafenamide (approved by EMA in 2018) is for children ages 12 years and older with body weight over 35 kg. Lamivudine is approved for children aged three years.

We would like to highlight the advantages of interferon-alpha and PegIFN-α for use in children compared with nucleoside and nucleotide analogues, which are the expected terminal duration of treatment and the absence of viral resistance. However, the use of interferons is associated with difficulties of administration – subcutaneous injections (three times a week for interferon-alpha or once a week for PegIFN-α) and a high risk of complications. AASLD recommends the use of PegIFN-α-2a in children over five years of age with chronic HBV infection. A meta-analysis showed that sustained response (combination of HBeAg seroconversion, serum HBV DNA undetectable with normalised ALT levels) and loss of HBsAg were significantly more common in children treated with IFN compared with untreated children [28]. Prognostic factors for successful IFN therapy are low serum HBV DNA levels, elevated ALT levels, younger age, female sex and active liver histological inflammation [32, 33].

In evaluating the efficacy of therapy, virological, serological, biochemical, and histological response to treatment is distinguished (Fig 1). This is assessed at a certain time of treatment and after the end of therapy. The following are indicative of the treatment efficacy: reduction of HBV DNA in serum to an undetermined level, elimination of HbeAg, normalisation of the ALT level.

Monitoring HBV infection in children, which includes assessment of the main characteristics of the course of chronic HBV infection before, during and after antiviral therapy, is of fundamental importance, both according to international recommendations and national guidelines. The frequency of assessment depends on the severity of liver fibrosis, the serological profile of the patient (HBeAg-positive or HBeAg-negative) and the ALT and HBV DNA levels (Fig 2). The ESPGHAN guidelines suggest the following monitoring plan for children with chronic HBV infection [14].

New strategies for the combination treatment of CHB, aiming to eliminate all replicative intermediates, including covalently closed-ring DNA (cccDNA) in the nucleus, are being investigated nowadays, raising the hope of important changes in the therapeutic strategy for HBV infection in non-HBV infections. [11,13,14].

## Conclusion

Summarising data from the guidelines for diagnosis and treatment of HBV infection presented by the major scientific international societies (ESPGHAN, AASLD, APASL, EASL), it can be stated that nowadays there are no studies that identify predictors of risk for HBV reactivation in children and adolescents and convincing evidence for the risks and benefits of specific strategic treatments. Thus, most of the consensus positions presented are based on data from multicentre studies in adults and on expert opinion.

It should be noted that despite the fact that vaccination of children against hepatitis B has been carried out in Ukraine since 2002, the problem of this disease remains quite acute. Due to the Standard of Medical Care for Childhood Viral Hepatitis B, approved in 2021 by the Order of the Ministry of Health of Ukraine, sick patients have the opportunity to use modern therapy strategies, namely direct antiviral drugs, which are purchased at the expense of the State Budget and distributed to the regions in accordance with this need. But, due to Russia's military aggression against Ukraine, there are difficulties with timely provision of drugs to children with HCV, who cannot interrupt treatment due to the health consequences. The protracted war in Ukraine has posed new challenges for doctors – adapting treatment and patient monitoring approaches, reducing the number of sick children lost from follow-up and timely prevention of HBV reactivation risks, and most importantly, the threat to the successful implementation of key objectives of the Strategy for the Prevention, Diagnosis and Treatment of Viral Hepatitis.
